# Droplet-based synthesis of homogeneous magnetic iron oxide nanoparticles

**DOI:** 10.3762/bjnano.9.226

**Published:** 2018-09-10

**Authors:** Christian D Ahrberg, Ji Wook Choi, Bong Geun Chung

**Affiliations:** 1 Research Center, Sogang University, Seoul, Korea; 2 Department of Mechanical Engineering, Sogang University, Seoul, Korea

**Keywords:** capillary reactor, droplet synthesis, magnetic nanoparticles, narrow size distribution

## Abstract

Nanoparticles have gained large interest in a number of different fields due to their unique properties. In medical applications, for example, magnetic nanoparticles can be used for targeting, imaging, magnetically induced thermotherapy, or for any combination of the three. However, it is still a challenge to obtain narrowly dispersed, reproducible particles through a typical lab-scale synthesis when researching these materials. Here, we present a droplet capillary reactor that can be used for the synthesis of magnetic iron oxide nanoparticles. Compared to conventional batch synthesis, the particles synthesized in our droplet reactor have a narrower size distribution and a higher reproducibility. Furthermore, we demonstrate how the particle size can be changed from 5.2 ± 0.9 nm to 11.8 ± 1.7 nm by changing the reaction temperature and droplet residence time in the droplet capillary reactor.

## Introduction

Due to their small size and large surface area, nanoparticles offer interactions with biological systems that classical bulk materials cannot provide [1]. Due to these special properties, nanoparticles have become of particular interest for a number of applications in information and energy storage [2], environmental studies [3], or in medicine [4]. In medical applications, a particular focus of research lies on the development of multifunctional nanomaterials, as they allow for the parallel treatment and diagnostic monitoring of a diseases and thus would help to reduce the costs of healthcare significantly [5]. For example, a composite nanomaterial has been developed as a photosensitizer in photothermal therapy (PTT), while also acting as a contrast agent for magnetic resonance imaging (MRI) [6]. Magnetic materials are of particular interest here, as they can be used for targeting [7], as contrast agents for MRI [8–9], or for magnetically induced thermotherapy [10]. Furthermore, they can be used for a combination of these functions [11–12]. However, batch synthesis on small scales often suffers from batch-to-batch reproducibility issues, and inhomogeneities of the chemical and thermal environment, making the controlled synthesis of these nanomaterials for research difficult [13].

Methods for synthesizing magnetic iron oxide nanoparticles can be divided into two categories: high temperature decomposition of iron precursors in organic solvents and the coprecipitation of iron salts under aqueous conditions. Thermal decomposition methods, as demonstrated by Rockenberger et al. [14] and Son et al. [15], have the advantage of producing highly uniform crystalline particles. However, complicated synthetic steps, toxic, and expensive reagents prevent this production of magnetic nanoparticles on the large scale. Coprecipitation methods, on the other hand, allow for the simple, scalable synthesis of magnetic iron oxide nanoparticles that can be dispersed in water without requiring further surface treatment [16]. Furthermore, the size of particles synthesized using coprecipitation can be controlled through the reaction temperature, as demonstrated by Mei and co-workers [17]. The quality of particles obtained using the coprecipitation method depends on rapid mixing of the iron precursor and alkaline solution [18–20]. Microfluidic systems are known for providing fast and reproducible mixing, while maintaining high control over the temperature of the sample [21]. Additionally, microfluidic devices can be operated with a high degree of automation allowing for continuous operation, while reaction parameters can be varied easily allowing for automatic screening of reaction conditions [22]. Therefore, microfluidic reactors are a promising option for lab scale production of nanoparticles. Continuous production of nanoparticles inside of a microfluidic reactor has been shown with CdSe nanoparticles, demonstrating precise control over the particle morphology [23]. Furthermore, a microfluidic steel reactor has been used by Laura Uson et al. for the synthesis of magnetic nanoparticles through a thermal decomposition reaction [24]. Microfluidic reactions can be operated using continuous flow or segmented flow. While single-phase continuous flow is simpler to operate, the laminar flow profile inside of the microfluidic channels leads to a residence time distribution of the reaction products. Contact of the reaction phase with the channel walls can further lead to fouling of the reactor. These problems can be prevented by introducing a second phase leading to the breaking up of one phase into several distinct droplets. Furthermore, chaotic advection inside of the droplets can increase the mixing performance of these devices [25]. Current microfluidic technology allows for the production of stable droplets in large numbers with high production frequencies [26]. Therefore, a number of particles have been synthesized in pinched flow microfluidic geometries, such as fluorescent silica particles [13], or titanium dioxide particles [27]. For these applications, the microfluidic devices were made from poly(dimethylsiloxane) (PDMS) following a soft-lithography fabrication method [28–29], or made by laser cutting from acrylic polymers [27]. Through these devices, it was possible to reduce reaction times significantly compared to batch reactions. Furthermore, in the case of spin-crossover particles, a 20-fold downsizing of particles compared to batch reactions could be observed [29]. Although these microfluidic methods offer advantages over classical batch synthesis, they require expertise in manufacturing and operation of microfluidic devices. To decrease the complexity of microfluidic devices, and to make the technology more accessible to untrained users, devices made from simple tubing and capillaries have been suggested [30]. Through these advantages provided by microfluidic droplet reactors, it should be possible to synthesize the particles with narrower size distributions and higher reproducibility compared to conventional batch synthesis [31]. In the specific case of the coprecipitation of iron oxide, nanoparticles inside of droplets should have significant benefits due to the fast mixing and thermal control compared to batch synthesis.

Here, we show a droplet capillary reactor for the synthesis of magnetic iron oxide nanoparticles using a coprecipitation reaction. Nanoparticles synthesized in the droplet reactor are compared to nanoparticles prepared through conventional batch synthesis in terms of particle size distribution and reproducibility. Furthermore, we demonstrate how the size of the synthesized particles can be adjusted by varying the reaction time in the droplet reactor or the reaction temperature.

## Experimental

### Fabrication of droplet capillary reactor

The droplet capillary reactor was made from a 100 cm long section of Tygon tubing (inner diameter = 0.51 mm, outer diameter = 1.52 mm, Sigma-Aldrich, USA) into which a through-hole was punched 15 cm from the end of the tubing using a small syringe needle. Two 5 cm long pieces of fused silica capillary (inner diameter = 100 µm, outer diameter = 360 µm, Supelco, USA) were inserted into the hole and arranged so that the two ends formed a 90° angle in the middle of the Tygon tubing. The holes were sealed with small amount of hot-melt adhesive. The remaining tubing after the junction was submerged in a water bath and the end was placed into a vial filled with water for sample collection. For droplet experiments the formation of droplets was recorded using a bright-field microscope (IX37, Olympus, Japan). Droplet diameters were manually measured using Image J (National Institute of Health, USA). For volume calculations, droplets were assumed to be of spherical shape if the droplet diameter was smaller than the diameter of the tubing, and of cylindrical shape for larger droplet diameters.

### Synthesis of magnetic iron oxide nanoparticles

Nanoparticles were synthesized using a coprecipitation reaction as previously described [17]. Briefly, two aqueous solutions were mixed. The first solution contained 0.06 M of FeCl_3_·6H_2_O (Alfa Aesar, USA) and 0.03 M of FeCl_2_∙4H_2_O (Sigma-Aldrich, USA) dissolved in water. The second solution was an aqueous solution of 4 M ammonia (Alfa Aesar, USA). The solutions were separately injected through the two fused silica capillaries of the droplet generator using syringe pumps with identical flowrates. Mineral oil (M5904, Sigma-Aldrich USA) with 0.075 vol % Triton X-100 (Samchun Chemical, Korea) and 1.75 vol % Abil EM 90 (Evonik Industrial, Germany) was injected through the central Tygon tubing, also using a syringe pump, forming the continuous phase. The water bath was heated to a temperature of 70 °C using a hot plate. At the end of the reactor tubing, droplets were collected in a vial filled with water. Exact experimental parameters regarding the comparison between batch and droplet synthesis (Table S1, Table S2), the effect of temperature (Table S3), and the effect of residence time on particle size (Table S4) can be found in Supporting Information File 1. For batch synthesis, analogous conditions were chosen (Table S2). Equal amounts of the two solutions were vigorously mixed in glass vial before being placed in a water bath at the same temperature (70 °C) and for the same time (11.1 min), as used for droplet experiments. After a predetermined time, the reaction was quenched with copious amounts of water. All experiments were repeated in independent experiments three times.

### Analysis of nanoparticles

The particles were collected from solution using a permanent magnet and the supernatant was removed. Afterwards, particles were repeatedly washed with water and separated using a permanent magnet until the supernatant remained clear. The remaining particles were freeze-dried overnight. The pure particles were resuspended in water at a concentration of 0.1 mg/mL and were subsequently transferred onto a transmission election microscopy (TEM) copper grid (Electron Microscopy Science, USA) by repeatedly dipping the grid in the previously prepared solution. TEM images were taken using a JEM-2100F (JEOL, Japan) and particle sizes were analyzed using Image J (National Institute of Health, USA). For all conditions, 100 particles were measured to determine the resulting size distribution. Zeta potentials of the particles suspended in DI water were determined using a Zetasizer Nano ZS (Malvern Panalytical, United Kingdom).

## Results and Discussion

### Fabrication of droplet capillary reactor

**Figure 1:**
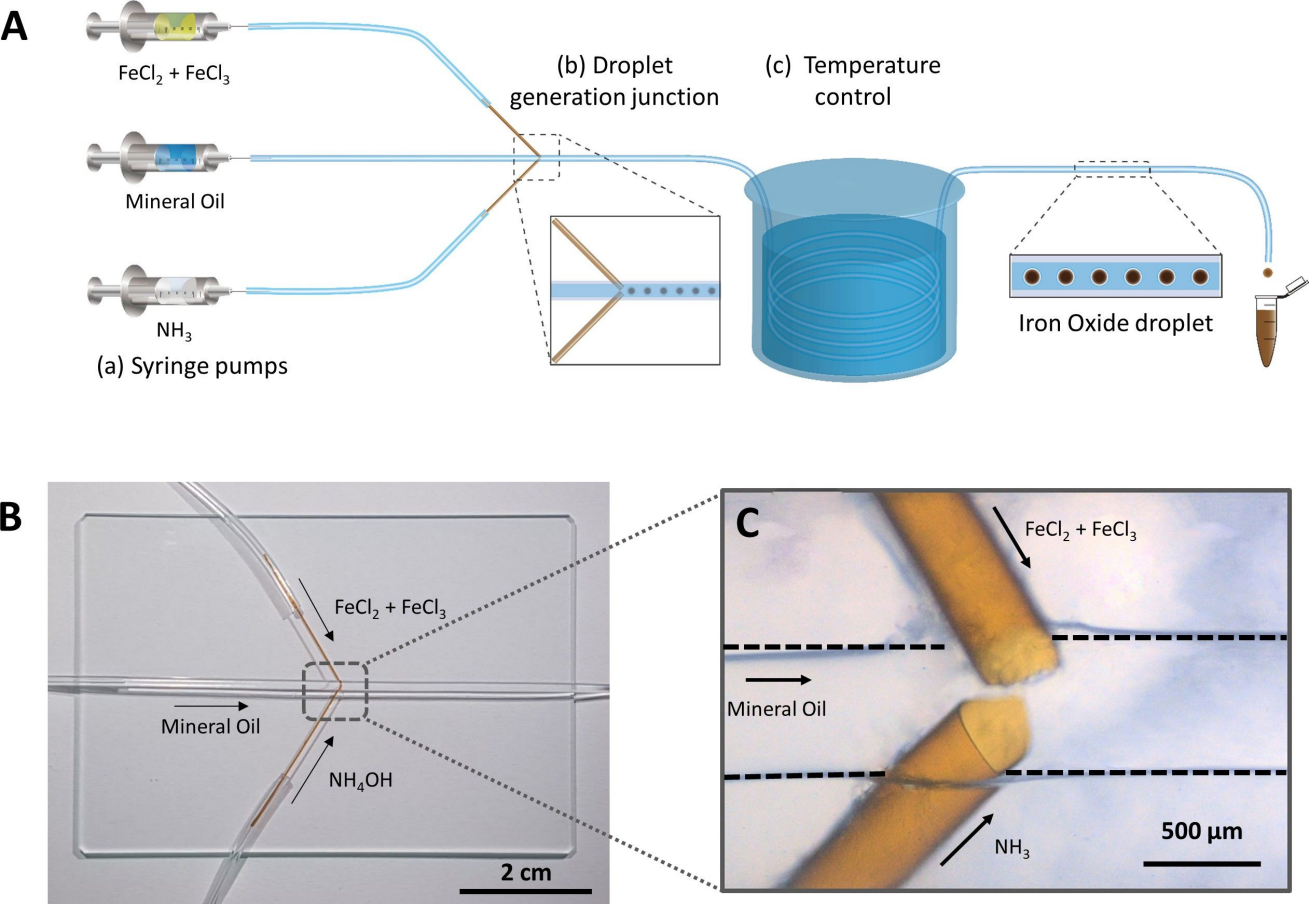
Schematic of the experimental setup showing the three syringe pumps for the reagent solutions and continuous oil phase, the droplet generation junction, the water bath for heating, and collection of droplets in a vial (A). Photograph of the droplet generation junction (B), and microscope image of the droplet generation junction showing the alignment of the fused silica capillaries in the center of the Tygon tubing (C).

**Figure 2:**
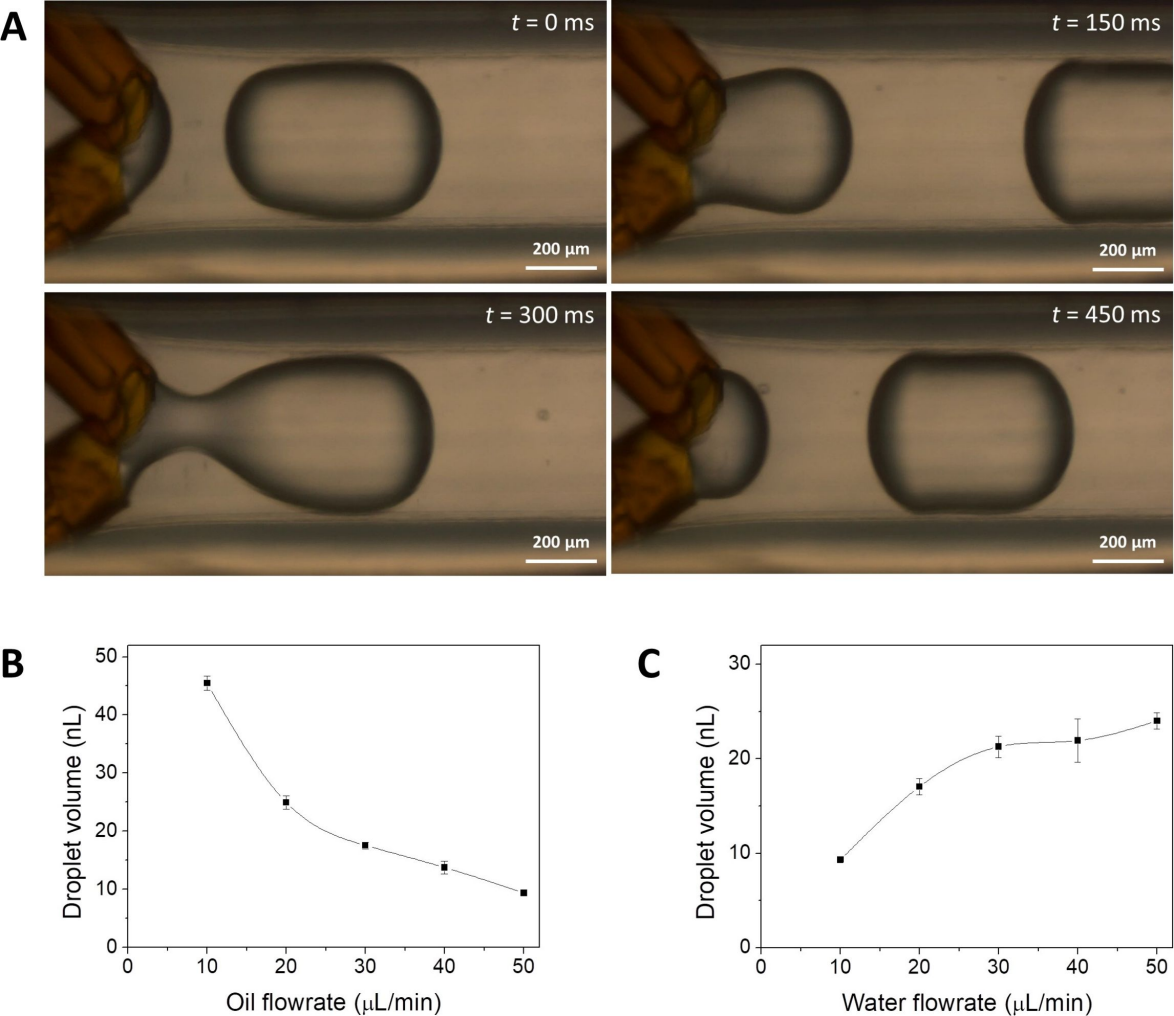
Time-lapse microscopy images of droplet generation inside the Tygon tubing (A). Graph of droplet volume as a function of oil flowrate at a water flowrate of 10 µL/min from both capillaries (B), and graph of droplet volume as a function of water flowrate at an oil flowrate of 50 µL/min (C). For the calculation of volumes 25 droplets were measured at each flowrate.

The droplet capillary reactor was made from Tygon tubing and fused silica capillaries. After the droplet generation, junction with a length of 85 cm of tubing was submerged in a water bath to obtain temperature control over the reaction (Figure 1). The generation of droplets by the device was tested. Through the central tubing, mineral oil was flowed, while an aqueous phase was injected through both fused silica capillaries, resulting in the formation of stable droplets within the tubing (Figure 2A). Through the close proximity, the two aqueous flows from the capillaries immediately merged to form one droplet. Small perturbations of the manual capillary alignment did not influence the formation of droplets, as long as the capillary tips remained in proximity of each other. If the flowrate for the oil phase is increased significantly above the range used here, the formation of individual droplets from each capillary can be observed. The volume of the droplets and their generation frequency could be manipulated by changing the flowrates of the aqueous or oil phase through the device (Figure 2B,C). As a cylindrical model negotiating the curvature of the front and back of the drops was used for volume calculation when the droplet diameter was larger than the tubing diameter, the calculated volumes overestimate the droplet volume slightly. To test the mixing of the two aqueous phases inside of the droplet, a colorless aqueous solution of FeCl_3_ was injected through one of the glass capillaries, while another colorless aqueous solution of KSCN was injected through the other fused silica capillary. Upon mixing of the two solutions, a red iron complex was immediately formed. In our experiments, droplets obtained a homogenous red color shortly after droplet formation, indicating fast mixing inside of the droplets required for the synthesis of iron oxide nanoparticles (Figure S1 in Supporting Information File 1, and Supporting Information File 2). After internal mixing the droplets showed a homogenous red color. To stabilize the droplets in the tubing, two surfactants (Abil EM 90 and Triton X-100) were added to the mineral oil phase. The concentration of the surfactants was chosen in such a manner that the droplets did not merge within the length of the tubing. However, once the droplets were collected in a water-filled vial, the aqueous droplets were immediately merged with the water phase, quenching the reaction occurring in the droplets.

### Synthesis of magnetic nanoparticles: comparison batch to droplet synthesis

**Figure 3:**
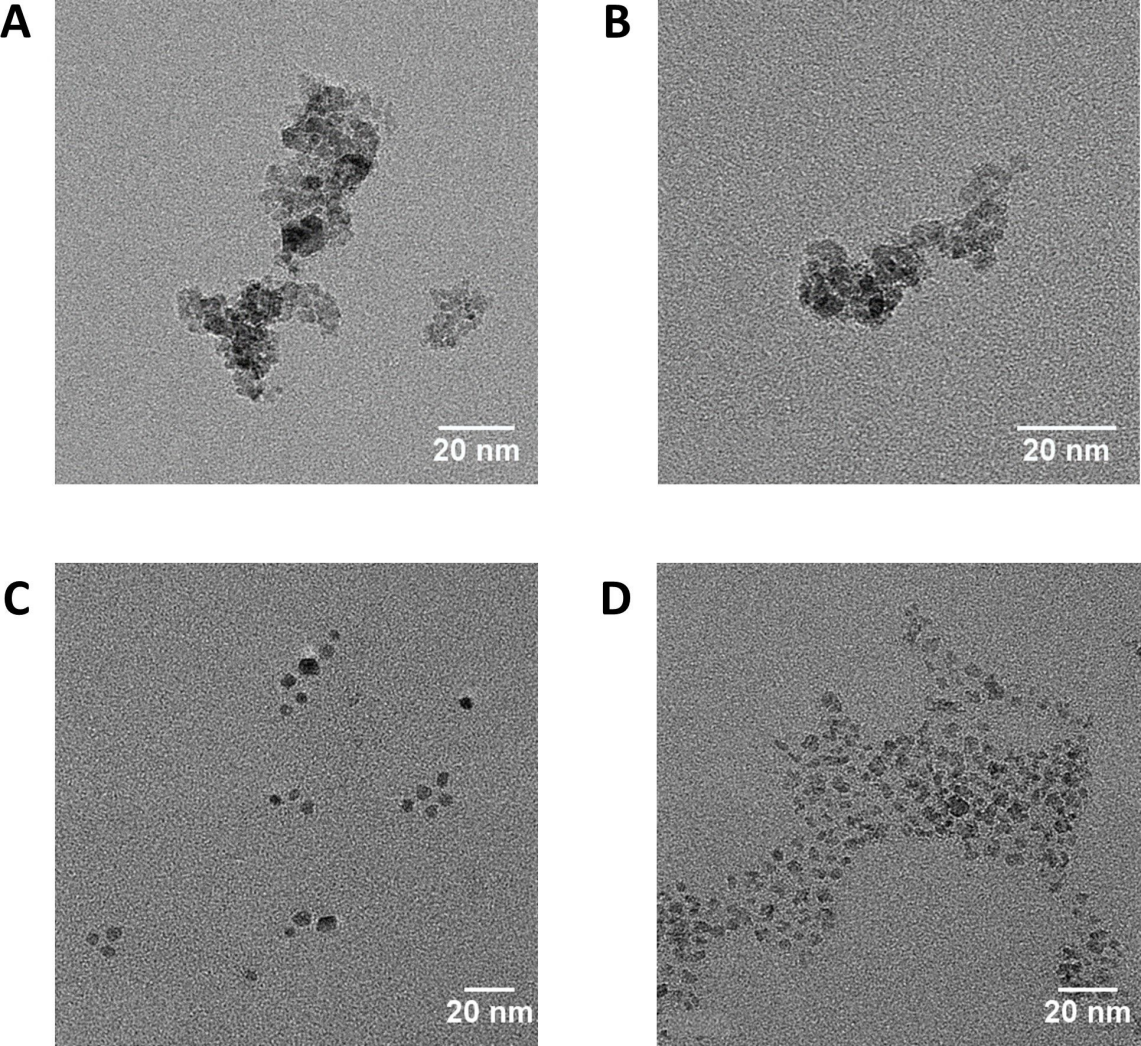
TEM images of nanoparticles synthesized by conventional batch synthesis and in droplet capillary reactors. TEM images of two examples of particles from two independent batch syntheses (A,B), and TEM images of two independent particle syntheses in droplet reactors (C,D).

In theory, nanoparticle synthesis in droplets should result in reproducible nanoparticles with a narrow size distribution, compared to particles synthesized through conventional batch synthesis. To evaluate this statement, magnetic iron oxide nanoparticles were synthesized in a batch reaction and in our droplet device. In droplet synthesis and in batch synthesis, mixing of the iron chloride precursor solution with the ammonia leads to the immediate formation of a dark brown precipitate. For both methods, the same reagent concentrations and reaction times were chosen. Following both synthesis methods, nanoparticles could be obtained (Figure 3). While the particles synthesized in a conventional batch reaction showed a wide dispersion and varying shapes in their TEM images (Figure 3A,B), iron oxide nanoparticles synthesized in droplets were more homogenous in size and shape (Figure 3C,D). To confirm the elemental composition of the nanoparticles energy-dispersive X-ray spectroscopy (EDS) was conducted after TEM imaging (Figure S2A,B in Supporting Information File 1), confirming that the particles consisted of Fe_3_O_4_. Moreover, the electron diffraction patterns were analyzed (Figure S2C,D in Supporting Information File 1), finding the same spinel crystalline planes as previously reported in literature for Fe_3_O_4_ nanopowders [32]. Hence, the synthesized particles are crystalline. Regarding the yield of the reaction both synthesis delivered comparable nanoparticle yields after washing and freeze-drying of the particles with 59.6 ± 21.7% yield for batch synthesis and 75.2 ± 8.8% yield for droplet-based synthesis. It should be noted here that the yield per reaction volume can be increased easily in batch reactions by increasing the iron precursor concentration. However, if the concentration is increased by more than one order of magnitude in droplet reactions, the fast precipitation of large amounts of nanoparticles could lead to the buildup of nanoparticle deposits on the fused silica capillaries in some cases and to a blocking of the capillaries. The batch particles showed a zeta potential of 20.2 ± 0.25 mV, and particles synthesized in droplets had a zeta potential of 16.9 ± 0.5 mV. The two measured potentials are in the same range, indicating a relative colloid stability of the particle solutions [33].

**Figure 4:**
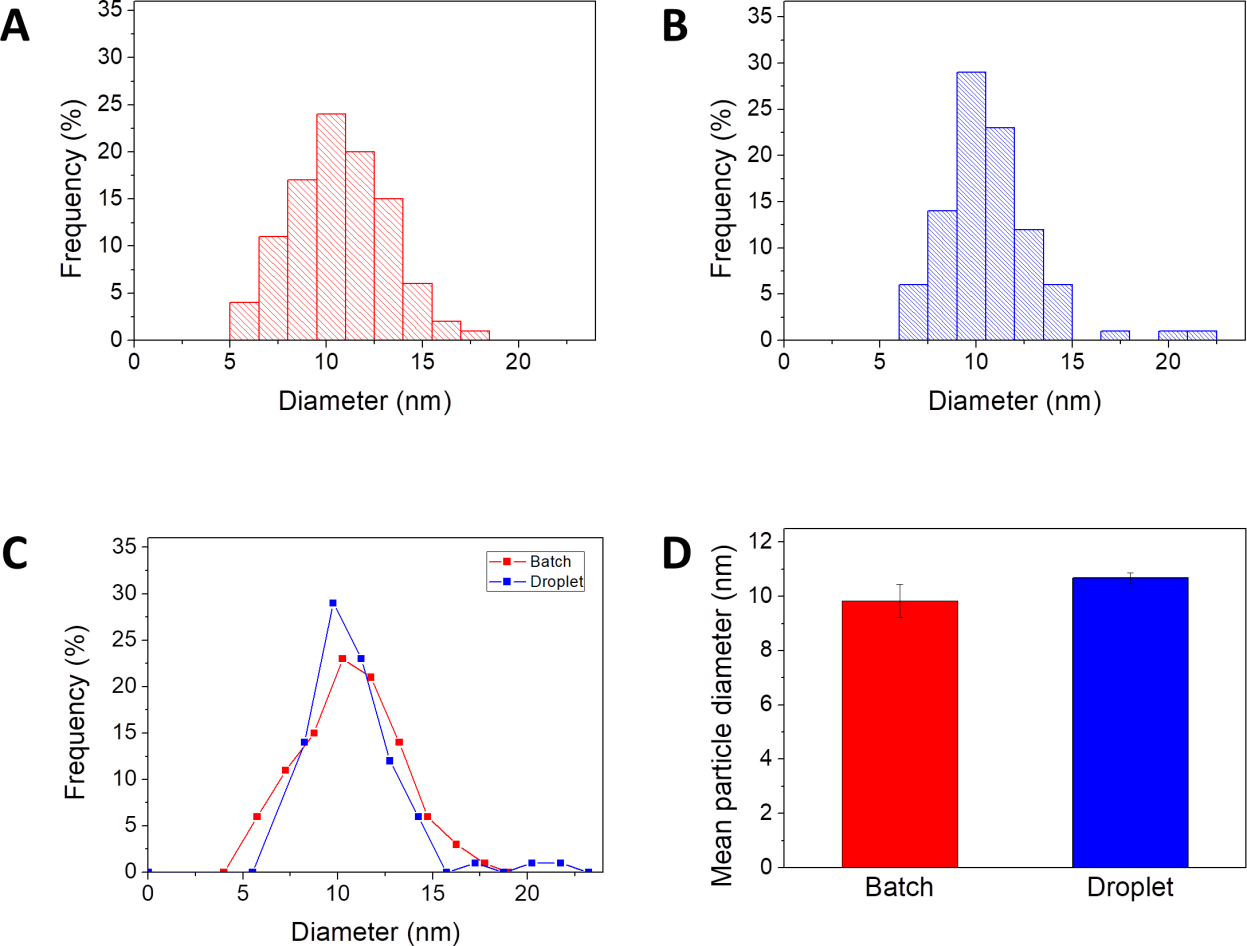
Histograms of magnetic nanoparticle size of particles synthesized in batch (A), and in droplets (B) under the same conditions. Overlay of the two particle distributions (C). Bar diagram showing the reproducibility of the reaction, three independent synthesis were conducted for each method and the mean particle diameter of the three synthesis is indicated by the bars (D).

To test the advantages of droplet synthesis over batch synthesis, particle size distributions were compared after reactions under the same conditions (Figure 4). The size of particles synthesized in a batch reactor and in droplets followed a normal distribution (Figure 4A,B) with an almost identical mean particle size (µ_batch_ = 10.6 ± 0.16 nm, µ_droplet_ = 10.5 ± 0.11 nm). However, comparing the spread of the particle size distributions, it can be seen that the batch-synthesized particles have a wider distribution (σ_batch_ = 2.4 ± 0.27 nm), compared to particles synthesized in droplets (σ_droplet_ = 1.8 ± 0.11 nm, Figure 4C). This confirms the earlier prediction that the continuous mixing inside of the droplets and the homogenous chemical and thermal environment should result in a better quality of nanoparticles. Furthermore, the reproducibility of particle synthesis was compared through three independent repetitions of each synthesis (Figure 4D). The average of the mean particle size of nanoparticles synthesized in three batch reactions was 9.9 ± 0.6 nm, while particles synthesized in three droplet synthesis had an average size of 10.6 ± 0.2 nm, as expected from previous experiments. The higher reproducibility of the droplet capillary reactor is due to the elimination of manual handling steps, such as mixing and quenching. In addition, the high degree of automation of droplet reactors further increases the reproducibility [34].

### Control of nanoparticle synthesis in droplets

**Figure 5:**
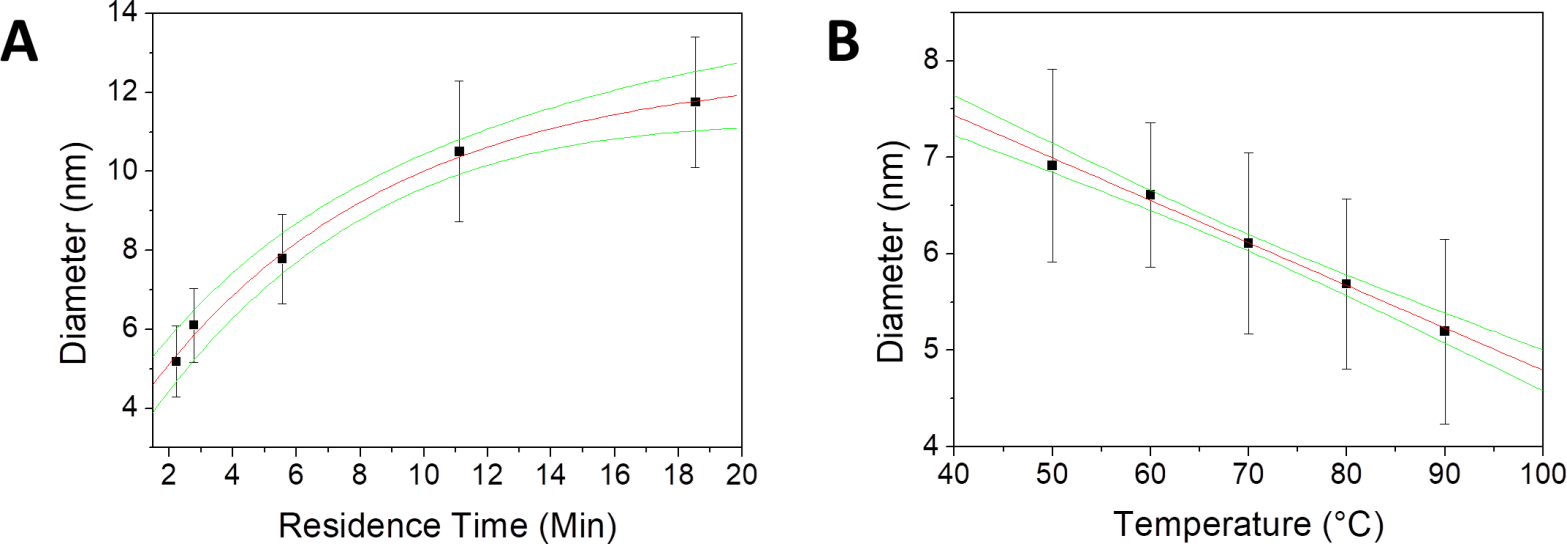
Analysis of particle sizes in droplet synthesis. Nanoparticle diameter as a function of the residence time of droplets in the reactor (A), and nanoparticle diameter as a function of the reaction temperature (B). In both graphs, the measured particle diameters are indicated by black squares, the standard deviation of the measured particle diameters is shown by black error bars. An exponential function was fitted in the case of (A) and a linear function was fitted for (B) (red lines). Green lines indicate the 95% confidence interval on the fitted functions. The R-square value for the exponential fit (A) is 0.986 and, respectively, 0.992 for the linear fit (B).

In a last set of experiments, it was evaluated how the size of particles synthesized in droplets can be influenced through the reaction parameters. For this, the residence time of droplets in the reactor was changed by varying the flow rate, further the reaction temperature was changed. A positive relationship between the mean particle size and the residence time of the droplets in the reactor could be observed (Figure 5A, Figure S3A,B in Supporting Information File 1). At short residence times (2–8 min), the effect of small changes in residence time on the mean particle diameter is quite large. At longer residence times, however, the effect becomes smaller due to depletion of reagents, and hence a slowdown of the reaction. Furthermore, the particle size distribution is widening with increasing residence time in the reaction, starting ta a standard deviation of 0.9 nm at a residence time of 2.2 min and increasing to 1.7 nm at a residence time of 18.5 min. Although classical theory for nanoparticle nucleation and growth predicts a narrowing of the particle size distribution with increasing reaction time [35–36], the opposite is observed in our experiments. This is probably due to classical models not including effects such as Ostwald ripening and, in our case more significant, the aggregation of nanoparticles, which can lead to the opposite effect [37]. The effect of reaction temperature on the nanoparticle size was further tested (Figure 5B, Figure S3C,D in Supporting Information File 1). It could be seen that with increasing temperature the mean diameter of the particles decreased linearly from 6.9 nm at 50 °C to 5.2 nm at 90 °C. In contrast to the experiments in which the droplet residence time was varied, the width of the distributions remained constant. This, most likely, is due to the formation of aggregates being strongly dependent on time and only weakly dependent on temperature. Hence, while changing the residence time of droplets allows for a wide range of particle sizes to be manufactured, changing the reaction temperature is more viable, as it does not affect the particle size distribution.

## Conclusion

Here, we show the synthesis of narrowly dispersed, magnetic iron oxide nanoparticles inside of a microfluidic droplet capillary reactor. Due to the advantages of this droplet capillary reactor, a narrower particle size distribution and higher reproducibility, compared to conventional batch synthesis, could be achieved. Furthermore, we demonstrated how the particle size could be manipulated by changing the reaction conditions of the droplet capillary reactor. The synthesis in droplet capillary reactors could be a valuable tool for lab scale synthesis of nanoparticles due to simple fabrication, easy operation, and higher reproducibility. In future research, the development of a multi-stage synthesis system would be of interest. Through this, larger nanoparticles could be synthesized by successive addition of reagents to the droplets, or core–shell particles could be made in a multi-step reaction within the droplets.

## Supporting Information

File 1 Additional experimental data.

File 2 Video showing droplet formation and mixing inside of the droplets.
Movie showing the droplet generation at an oil flow rate of 10 µL/min, and a flow rate of 10 µL/min from each capillary. A solution of iron(III) chloride was used from one capillary and of potassium thiocyanate from the other capillary. Upon mixing of the two solutions are red complex was immediately formed. It can be seen that both capillaries feed into the same droplets. After adequate mixing the absorption of the droplets was 43.5 ± 0.19 (arbitrary units).
