# Supplementary material for: Droplet-based synthesis of homogeneous magnetic iron oxide nanoparticles
Source: Beilstein J Nanotechnol. 2018 Sep 10;9:2413–20. doi: 10.3762/bjnano.9.226 (PMC6142730; doi:10.3762/bjnano.9.226)
Supplement: File 1 — Additional experimental data. [file Beilstein_J_Nanotechnol-09-2413-s001.pdf]

# Supporting Information

for

# Droplet-based synthesis of homogeneous magnetic iron oxide nanoparticles

Christian D. Ahrberg[‡1], Ji Wook Choi[‡2] and Bong Geun Chung*[2]

Address: [1]Research Center, Sogang University, Seoul, Korea and [2]Department of Mechanical Engineering, Sogang University, Seoul, Korea

Email: Bong Geun Chung* - bchung@sogang.ac.kr

* Corresponding author

‡ Equal contributors

## Additional experimental data

**Table S1:** Droplet reaction conditions for comparing droplet and batch syntheses.

| | value | unit |
|---|---|---|
| concentration $FeCl_3·6H_2O$ (solution A) | 0.06 | M |
| concentration $FeCl_2·4H_2O$ (solution A) | 0.03 | M |
| concentration $NH_3$ (solution B) | 4 | M |
| flow rate (solutions A and B, mineral oil) | 5 | µL/min |
| temperature | 70 | °C |
| residence time | 11.1 | min |

**Table S2:** Batch reaction conditions droplet and batch syntheses.

| name | value | unit |
|---|---|---|
| concentration $FeCl_3·6H_2O$ (solution A) | 0.06 | M |
| concentration $FeCl_2·4H_2O$ (solution A) | 0.03 | M |
| concentration $NH_3$ (solution B) | 4 | M |
| amount (solutions A and B), ratio 1:1 | 100 | µL |
| temperature | 70 | °C |
| reaction time | 11.1 | min |

**Table S3:** Droplet reaction conditions used for analyzing the effect of temperature on the particle size.

| name | value | unit |
|---|---|---|
| concentration $FeCl_3{\cdot}6H_2O$ (solution A) | 0.06 | M |
| concentration $FeCl_2{\cdot}4H_2O$ (solution A) | 0.03 | M |
| concentration $NH_3$ (solution B) | 4 | M |
| flow rate (solutions A and B, mineral oil) | 20 | µL/min |
| temperature | 50/60/70/80/90 | °C |
| residence time | 2.8 | min |

**Table S4:** Droplet reaction conditions used for analyzing the effect of residence time on the particle size.

| name | value | unit |
|---|---|---|
| concentration $FeCl_3 \cdot 6H_2O$ (solution A) | 0.06 | M |
| concentration $FeCl_2 \cdot 4H_2O$ (solution A) | 0.03 | M |
| concentration $NH_3$ (solution B) | 4 | M |
| flow rate (solutions A and B, mineral oil) | 3/5/10/20/25 | µL/min |
| temperature | 70 | °C |
| residence time | 18.5/11.1/5.6/2.8/2.2 | min |

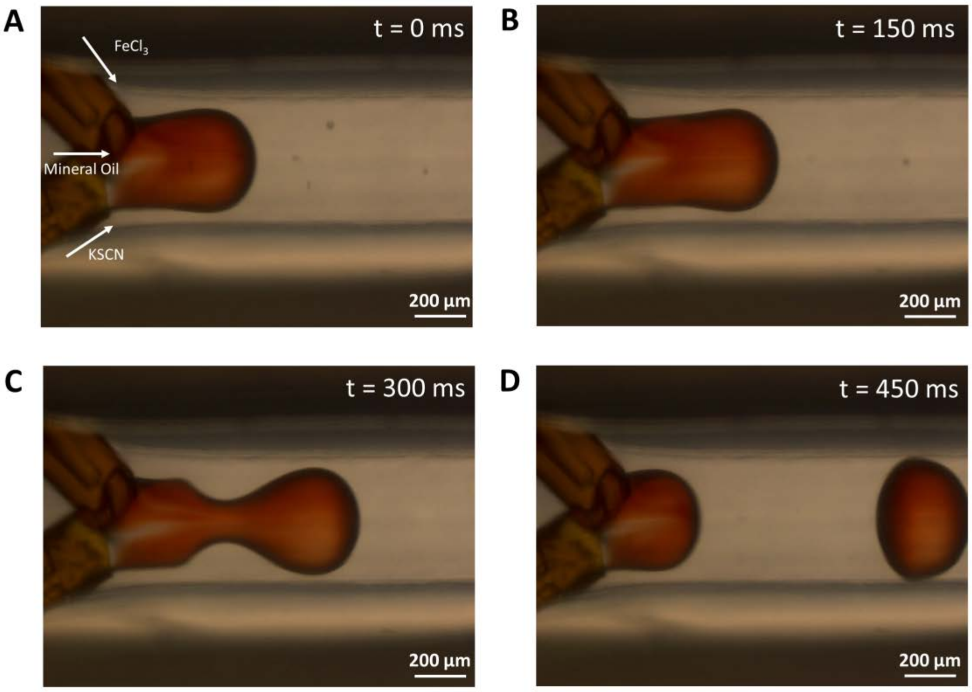


**Figure S1:** Microscope time-lap images illustrating mixing within the droplets taken at $t$ = 0 ms (A), $t$ = 150 ms (B), $t$ = 300 ms (C), and $t$ = 450 ms (D). For this experiment a colorless solution of $FeCl_3$ is flowed through the capillary coming from the top, and another colorless solution of KSCN is flowed through the capillary coming from the bottom. Upon mixing, a red iron complex is formed immediately ($FeCl_3$ + 3KSCN → $Fe(SCN)_3$ + 3 KCl), indicating mixing by the color change. Flow rates are 10 µL/min for the aqueous solution from the capillaries and 10 µL/min for the mineral oil, respectively.

A

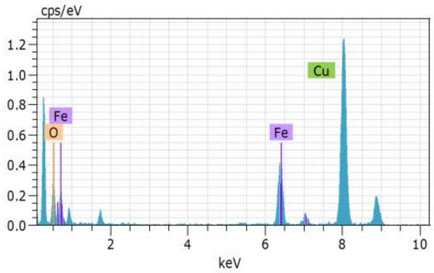


B

| Element | At% |
|---|---|
| Oxygen | 59.28 |
| Fe | 40.72 |

C

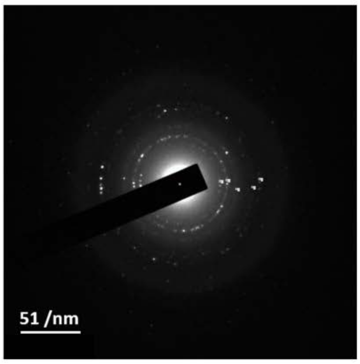


D

| Spot No. | D-Spacing [Å] | Spinel crystalline plane |
|---|---|---|
| 1 | 2.9 | 2 2 0 |
| 2 | 2.5 | 3 1 1 |
| 3 | 2.1 | 4 0 0 |
| 4 | 1.6 | 5 1 1 |
| 5 | 1.5 | 4 4 0 |

**Figure S2:** Energy-dispersive X-ray spectroscopy (EDS) of nanoparticles synthesized in droplets, recorded EDS spectrum of the particles (A). The large copper peak originates from the copper grid used for sample preparation. Elemental composition of the nanoparticles extracted from EDS spectrum (B), confirming that the particles consist of $Fe_3O_4$. The electron diffraction pattern of the particles confirms the crystallinity of the nanoparticles (C). Table of *d*-spacing values extracted from electron diffraction pattern with the corresponding spinel crystalline planes indicated (D).

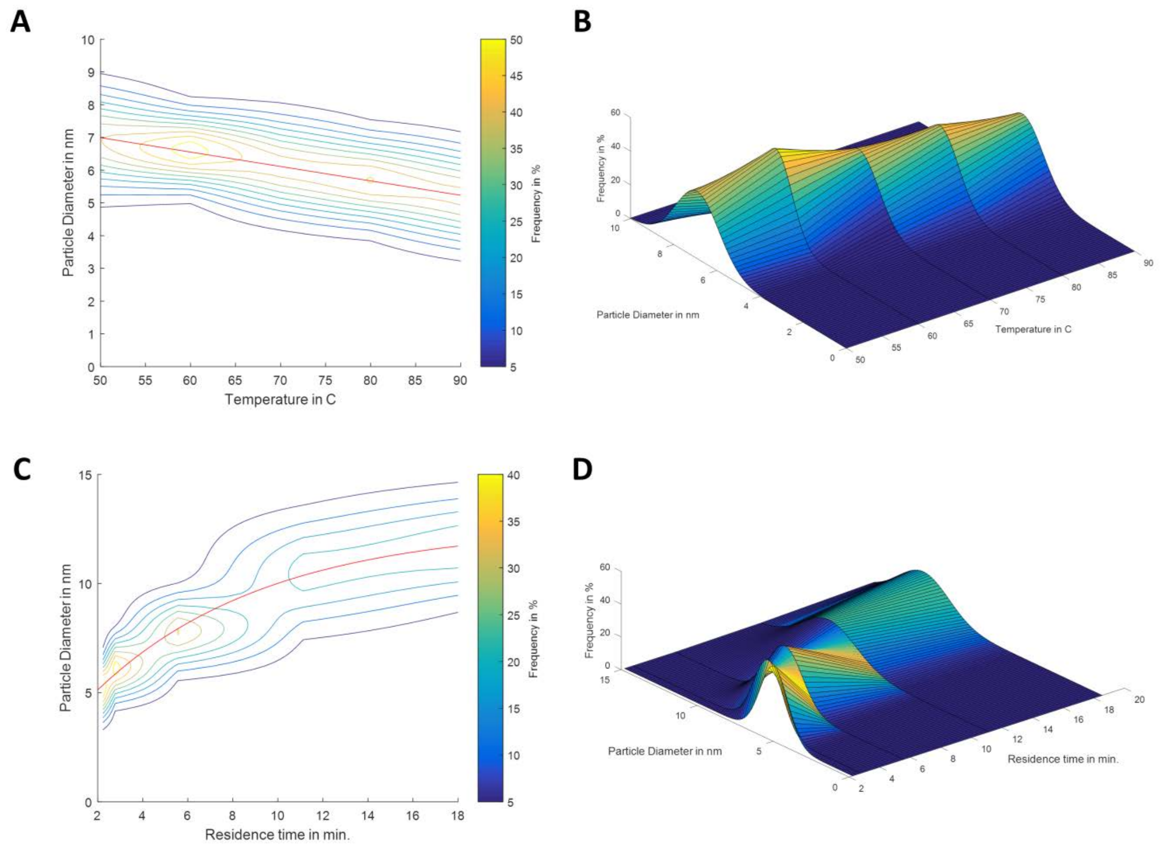


**Figure S3:** Contour (A) and surface plot (B) of the particle size distributions dependence on temperature, and contour (C) and surface plot (D) of the particle size distributions dependence on droplet residence time. In both contour plots, the fitted liner/exponential function is indicated by a red line.
